# The ubiquitin‐proteasome system and autophagy as guardians of the cellular proteome

**DOI:** 10.1002/1873-3468.70371

**Published:** 2026-05-28

**Authors:** Ivan Dikic

**Affiliations:** ^1^ Institute of Biochemistry II Goethe University Medical Faculty Frankfurt am Main Germany

**Keywords:** autophagy, ER quality control, phase separation, proteasome, proteostasis, ubiquitin

## Abstract

Maintaining a functional proteome is essential for cellular health and organismal longevity. Disruption of proteostasis is a hallmark of aging and a central driver of diverse pathologies, including neurodegeneration, cancer, and metabolic disease. The ubiquitin–proteasome system (UPS) and autophagy represent the two principal degradative pathways safeguarding proteome integrity, particularly under conditions of stress. While historically viewed as mechanistically distinct, it is now clear that UPS and autophagy operate as an interconnected and adaptive network. This Perspective discusses three core principles that govern their coordination: (1) a shared molecular language of ubiquitin signals and shuttle proteins that determines cargo routing; (2) spatial compartmentalization through organelle‐specific quality control modules and phase‐separated degradation hubs; and (3) temporal regulation by stress‐responsive signaling pathways that reprogram proteolytic output. Understanding this dynamic partnership not only reveals fundamental organizing principles of cellular homeostasis but also identifies new therapeutic nodes for diseases driven by proteostasis collapse.

## Graphical abstract

This Perspective covers the three principles governing the crosstalk between the ubiquitin‐proteasome system and autophagy in cellular proteostasis: (1) a shared ubiquitin code routing substrates via shuttle factors or autophagy receptors; (2) spatial compartmentalization into phase‐separated degradation hubs and organelle‐specific modules (exemplified by the endoplasmic reticulum); and (3) temporal regulation by stress signaling, ensuring adaptive proteostasis.

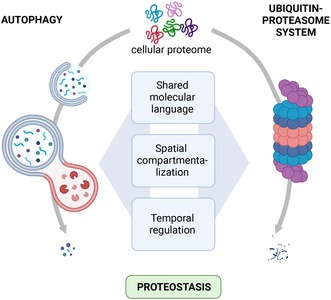

## Abbreviations

**AAA+ ATPase**, ATPases Associated with diverse cellular Activities

**AMFR**, Autocrine motility factor receptor

**ATF4**, Activating transcription factor 4

**ATL3**, Atlastin 3

**BAG**, Bcl2‐associated athanogene

**BiP**, Binding immunoglobulin protein

**BNIP3L/NIX**, BCL2 interacting protein 3 like

**CALCOCO1**, Calcium binding and coiled‐coil domain 1

**CCPG1**, Cell cycle progression 1

**CHIP**, C‐terminus of HSC70‐interacting protein

**CMA**, Chaperone‐mediated autophagy

**DUB**, Deubiquitinating enzyme

**E1**, Ubiquitin‐activating enzyme

**E2**, Ubiquitin‐conjugating enzyme

**E3**, Ubiquitin ligase.

**ER**, Endoplasmic reticulum

**ERAD**, ER‐associated degradation

**ERES**, ER exit sites

**ERLAD**, ER‐to‐lysosome‐associated degradation

**ER‐phagy**, ER‐selective autophagy

**FAM134B**, Family with sequence similarity 134 member B

**FIP200**, FAK family kinase‐interacting protein of 200 kDa

**GABARAP**, Gamma‐aminobutyric acid receptor‐associated protein

**HRD1**, HMG‐CoA reductase degradation protein 1

**HSF1**, Heat shock factor 1

**HSP**, Heat shock protein

**ISR**, Integrated stress response

**K48**, Lysine 48‐linked ubiquitin chain

**K63**, Lysine 63‐linked ubiquitin chain

**LC3**, Microtubule‐associated protein 1 light chain 3

**LIR**, LC3‐interacting region

**LLPS**, Liquid–liquid phase separation

**MARCH6**, Membrane‐associated RING‐CH protein 6

**NBR1**, Neighbor of BRCA1 gene 1

**NDP52**, Nuclear dot protein 52

**NPC1**, Niemann–Pick disease type C1 protein

**NRF1**, Nuclear factor erythroid 2‐related factor 1

**OPTN**, Optineurin

**PSG**, Proteasome storage granule

**PSMD14**, Proteasome 26S subunit, non‐ATPase 14 (Rpn11)

**Rad23**, Radiation‐sensitive protein 23

**RNF185**, Ring finger protein 185

**Rpn1**, Regulatory particle non‐ATPase subunit 1

**RTN3**, Reticulon 3

**sHSP**, Small heat shock protein

**TAX1BP1**, Tax1‐binding protein 1

**TBK1**, TANK‐binding kinase 1

**TDP‐43**, TAR DNA‐binding protein 43

**TEX264**, Testis‐expressed protein 264

**TFEB**, Transcription factor EB

**UBA**, Ubiquitin‐associated domain

**UBD**, Ubiquitin‐binding domain

**Ubl**, Ubiquitin‐like domain

**UCHL5**, Ubiquitin C‐terminal hydrolase L5

**ULK1**, Unc‐51‐like autophagy activating kinase 1

**UPS**, Ubiquitin–proteasome system

The timely removal of damaged, misfolded, or superfluous proteins is key to cellular proteostasis. Failures in protein disposal mechanisms lead not only to loss of protein function but to the accumulation of toxic protein species and dysfunctional organelles, driving aging and a wide range of diseases including neurodegeneration, cancer, and metabolic disorders [1, 2]. Two major proteolytic pathways maintain proteostasis: the Ubiquitin‐Proteasome‐System (UPS) and autophagy. The UPS typically handles the rapid turnover of short‐lived regulatory and misfolded proteins via the 26S proteasome—a barrel‐shaped 20S catalytic core capped by 19S regulatory particles that mediate substrate unfolding and translocation. Conversely, autophagy manages the lysosomal degradation of bulky cargo, such as protein assemblies and damaged organelles, through their sequestration into double‐membraned autophagosomes. Besides unspecific bulk autophagy, this process can selectively target specific cellular structures and organelles, for example, the ER (ER phagy), mitochondria (mitophagy), ribosomes (ribophagy), etc. (reviewed in [1, 3, 4, 5]). Although conceptually separated, the UPS and autophagy share molecular components, communicate through ubiquitin‐based signaling, and dynamically compensate for one another. They form a unified proteostasis network whose output is continuously rewired by ubiquitin signals, spatial organization, and stress‐induced remodeling. This Perspective outlines the fundamental principles underlying this integration, emphasizing how cells coordinate proteolytic flux to maintain homeostasis under fluctuating physiological demands.

## A shared molecular language—ubiquitin and beyond

### The ubiquitin routing code

**Fig. 1:**
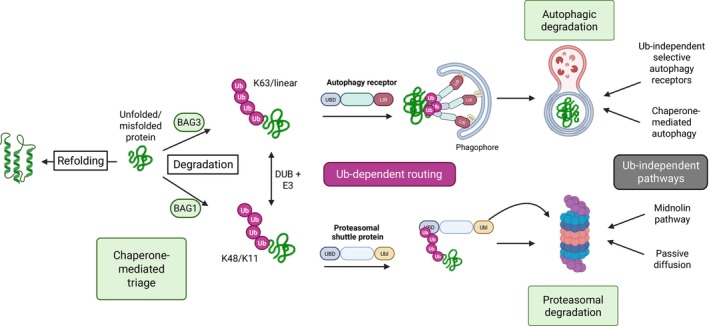
Integrated proteostasis network connecting UPS and selective autophagy. Misfolded or superfluous proteins are triaged by chaperones toward refolding or degradation (left). Ubiquitin modifications serve as a shared routing language. K48/K11‐linked chains favor proteasomal turnover via shuttle factors (Rad23, ubiquilin, p62), whereas K63/linear chains promote recruitment of autophagy receptors (p62, NDP52, OPTN) that connect cargo to LC3‐positive phagophores. DUB/E3‐mediated ubiquitin chain editing allows rerouting. Ubiquitin‐independent entry points exist for both pathways (20S proteasome diffusion, midnolin‐pathway, cargo‐embedded ER‐phagy receptors, chaperone‐mediated autophagy) (right).

Both UPS and autophagy rely on ubiquitin as a signal for cargo recognition, suggesting that evolution has converged on a common logic for selective substrate routing (Fig. 1). Substrate targeting relies on ubiquitination, a hierarchical enzymatic cascade involving E1 activating enzymes, E2 conjugating enzymes, and E3 ligases, which provide specificity [6]. Ubiquitination can give rise to ubiquitin chains of diverse topologies and lengths, encoding a broad repertoire of ubiquitin signals that may direct distinct outcomes [7]. Whereas K48‐ and K11‐linked chains typically favor proteasomal targeting [8, 9], K63‐linked or linear ubiquitin architectures often target substrates toward selective autophagy [10, 11]. This requires the accurate interpretation of the ubiquitin code.

The selective recognition and delivery of the ubiquitinated clients is orchestrated by specialized molecular adapters. In the UPS, shuttle proteins such as Rad23 and ubiquilins (Dsk2 in yeast), which contain both ubiquitin‐binding domains (UBDs) and proteasome‐interacting motifs, capture substrates and link them to the 19S regulatory particle [12]. Similarly, selective autophagy receptors like p62, NBR1, OPTN, and NDP52 act as bridges for bulky cargo; they combine UBDs to recognize tagged substrates with LC3‐interacting regions (LIRs) that anchor the cargo to the burgeoning autophagosomal membrane [13].

Importantly, ubiquitin‐dependent routing is highly dynamic. Deubiquitination enzymes can reverse ubiquitination, allowing the editing of ubiquitin chains on cargo to alter substrate fate [14, 15]. Furthermore, certain shuttle proteins and autophagy receptors can switch function and promote either proteasomal turnover or autophagic capture depending on their domain accessibility, oligomeric state, post‐translational modification, local concentration, and the type of ubiquitin modification attached to the substrate [16, 17]. A prime example for this phenomenon is p62. Under basal conditions, p62 is unphosphorylated at Serine 403 (S403), keeping its ubiquitin‐associated domain (UBA) in a low‐affinity state. This favors monomeric p62, which uses its PB1 domain to dock onto the 26S proteasome and deliver small, soluble K48‐ubiquitinated cargos, acting as a proteasomal shuttle [18, 19]. However, under stress conditions, S403 becomes phosphorylated (e.g., by TBK1 or ULK1), drastically increasing the UBA domain's affinity for both K48‐ and K63‐linked ubiquitin chains. This promotes p62 oligomerization and exposes the LIR motif leading to the recruitment of LC3 and directing cargo to autophagy [6, 7]. Thus, the same molecular adapters can toggle between degradation pathways, and this plasticity is rooted in their composite domain architectures.

### Ubiquitin‐independent entry points

Despite the central role of ubiquitin, this reliance is not exclusive. Both the proteasome and selective autophagy possess ubiquitin‐independent entry points, which need to be integrated into the proteostasis network (Fig. 1). Several selective autophagy pathways rely on other types of marks, including certain lipids [20] or sugar‐based modifications [21], or the receptor is an integral part of the cargo itself such as FAM134B [22, 23], RTN3 [24], or BNIP3L/NIX [25]. Similarly, potentially over 20% of the proteome can undergo proteasomal degradation independent of ubiquitination [26, 27, 28]. A subset of them, mostly intrinsically disordered, oxidized, or misfolded proteins, can passively diffuse directly into the catalytic chamber of the 20S proteasome, which can function without the 19S cap. In addition, many nuclear proteins, including various stimulus‐responsive critical transcription factors such as EGR1, Fos, NR4A1, IRF4, NeuroD1, and CBX4, have been shown to be subjected to ubiquitin‐independent degradation via the midnolin‐proteasome pathway [29]. Midnolin harbors three domains that act in concert to mediate degradation of these proteins by the 26S proteasome: a C‐terminal alpha‐helix that robustly interacts with the Rpn1 subunit of the 19S cap, a Catch domain interacting with the substrate, and an N‐terminal Ubl‐domain binding to PSMD14/Rpn11 [30]. Thus, midnolin repurposes the proteasomal components typically engaged by ubiquitinated substrates. It remains to be seen whether a cytosolic counterpart of midnolin will be discovered in the future.

### Molecular chaperones at the interface of UPS and autophagy

Crucially, degradation decisions—whether ubiquitin‐dependent or independent—rarely occur in isolation but are often preceded and shaped by the action of molecular chaperones, which act as the primary gatekeepers of protein quality control [31, 32]. Chaperones, including ATP‐dependent HSP70/40, HSP90, and the ATP‐independent small heat shock proteins (sHSPs), triage nascent or stress‐denatured proteins toward refolding, disaggregation, or degradation [32, 33]. The chaperone network directly interfaces with both UPS and autophagy by enabling substrate recognition, maintaining clients in a degradation‐competent state and preventing aberrant phase transitions that lead to solid aggregation [34, 35, 36]. In the case of chaperone‐mediated autophagy, they directly participate in the degradation of their clients [37]. Thus, the folding network defines the crucial window within which UPS and autophagy can productively act.

Moreover, chaperones do not merely hand over substrates but can be active regulators of the UPS–autophagy switch. A paradigmatic example is the BAG family of chaperones. While BAG1 promotes K48‐linked ubiquitination and proteasomal degradation via HSP70–CHIP complexes, BAG3 redirects misfolded clients to autophagy by engaging HSPB8 and activating the HSPB8–HSP70–HSPBP1 disaggregation machinery required for autophagic removal of aggregates (aggrephagy) [32, 36, 38, 39, 40]. In response to stress signaling and aging, there is an expression switch from BAG1 to BAG3 favoring autophagy when proteasome capacity is saturated [41].

## Spatial compartmentalization: Degradation hubs and organelle‐specific proteostasis

**Fig. 2:**
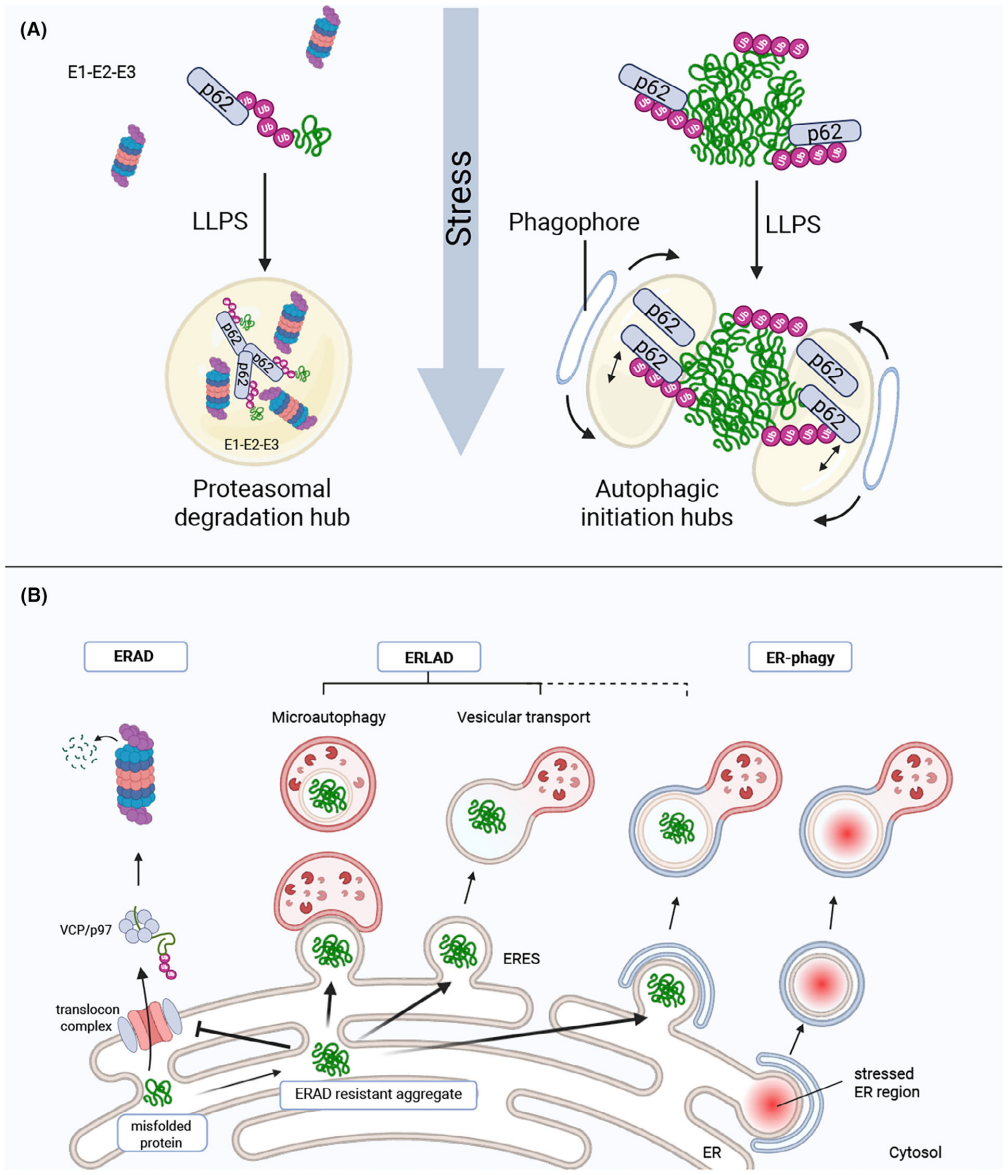
Spatial organization of degradation hubs and ER‐associated pathway switching. (A) Cells localize proteolytic activity into discrete microenvironments via liquid–liquid phase separation (LLPS). Proteotoxic stress induces formation of phase‐separated proteasomal condensates enriched in proteasomes, shuttles, and ubiquitinated substrates. In parallel, selective autophagy initiation hubs assemble directly on cargo surfaces. Formation of these hubs requires autophagy receptor mobility allowing the receptors to cluster and recruit scaffolding proteins such as FIP200/Atg11 to induce phagophore formation. (B) At the ER, misfolded proteins are preferentially cleared by ERAD through ubiquitination and proteasomal extraction (left). When ERAD is saturated or clients are ERAD‐resistant, ER‐phagy and ERLAD pathways reroute aggregated or ERAD‐resistant clients to lysosomes via specialized ER subdomains (e.g., ER exit sites (ERES), ER–lysosome membrane contact sites, or three‐way tubular junction), ensuring compartment‐specific proteostasis resilience. Moreover, ER phagy can eliminate stressed or superfluous ER subdomains (illustrated by red sphere) along with associated proteins through membrane remodeling (right).

Beyond biochemical signals, spatial organization has emerged as a decisive layer of proteostasis regulation (Fig. 2). Subcellular localization, together with local physicochemical conditions, strongly influences when and where the UPS or autophagy is engaged. This is particularly apparent at organelles, where differences in membrane architecture, luminal pH, redox state, or local ROS production impose distinct quality control demands (reviewed in [42, 43, 44]). Apart from membrane‐bound compartments, cells also exploit biomolecular condensates as organizing principles of proteolysis (Fig. 2A). These membrane‐less assemblies form through liquid–liquid phase separation (LLPS) or percolation transitions, depending on the molecular grammar involved (here, we use the terms “phase separation” and “condensates” in a broad sense to encompass these related behaviors, while noting that the precise mechanistic distinctions among them remain an active area of investigation and debate). Condensate‐driven compartmentalization can concentrate proteolytic machinery, substrates, and regulatory factors into discrete degradation microenvironments [45, 46, 47] , and has emerged as an important conserved strategy, from yeast to mammals, to fine‐tune proteostasis output in space and time.

### Proteasomal condensates as local degradation hot spots

Various cellular stresses can induce the formation of condensates that concentrate active proteasomes [46, 48, 49]. Some condensates require the oligomerization of shuttle factors such as p62, Rad23, and Dsk2 along with K48‐linked ubiquitin chains, thereby linking the chemical ubiquitin signal on the cargo to biophysical drivers of proteasomal degradation [12, 45]. Others are formed by the chaperone Bcl2‐associated athanogene 2 (BAG2) [50]. BAG2‐induced proteasomal granules are devoid of ubiquitin. They appear to form directly at the location of BAG2's client proteins, for example, on microtubules as with Tau protein, and promote ubiquitin‐independent degradation via the 20S proteasome.

The functional importance of distinct degradation “hot spots” is exemplified by p62‐dependent proteasomal condensates, which act as degradation hubs enriched in proteasomes and specific E3 ligases/DUBs in both the nucleus and cytosol [51]. Despite sharing a core mechanism, their biological outcomes may diverge sharply due to compartment‐specific substrate preferences and regulatory components: nuclear p62 condensates promote degradation of the oncoprotein c‐Myc and, through recruitment of the deubiquitinase USP7, stabilize the tumor suppressor p53. In the cytosol, p62 condensates accelerate MDM2‐mediated degradation of p53, lowering its steady‐state levels. Consequently, in the specific models studied, nuclear p62 condensates suppressed tumor growth (mainly by degrading c‐Myc and stabilizing p53), whereas cytosolic p62 condensates promoted tumor growth (by accelerating p53 degradation). Whether this dichotomy holds across different cancer types and genetic backgrounds (especially those with mutant or absent p53) remains to be investigated [51]. This divergence suggests that therapeutic strategies could focus on controlling p62's subcellular localization or its propensity to form phase‐separated degradation hubs.

However, not all proteasome clusters function as degradative hubs. In yeast, cytosolic condensates known as Proteasome Storage Granules (PSGs) form in adaptation to glucose starvation or quiescence [52]. PSGs are thought to protect (inactive) proteasomes from non‐selective autophagy, ensuring that vital proteolytic machinery is preserved under conditions of low energy availability. To do so, 26S proteasomes are packed into PSGs through a hierarchical, stepwise assembly process that results in a rigid, paracrystalline lattice [52]. This packaging is a direct, structural response to energy limitation and can be quickly reversed upon glucose replenishment because of the rigid but fragile nature of the paracrystals.

### Phase‐separated initiation hubs as organizing centers of selective autophagy

Autophagic processes are also closely connected to LLPS‐mediated spatial organization. Recent work proposes that selective autophagy initiation relies on phase‐separated receptor hubs forming directly on cargo surfaces [53]. A central aspect of this model is the mobility of autophagy receptors on the surface of a given cargo. This receptor mobility can be achieved by low‐affinity interactions with the cargo, such as through Ub‐UBD interactions or by phase separation of membrane‐less cargo. The mobile, cargo‐bound receptors then recruit the autophagy scaffolding protein FIP200 (or Atg11 in yeast), leading to its LLPS and the formation of multiple “initiation hubs” on the cargo periphery. These hubs recruit other early autophagy factors like Atg1/ULK1 and coalesce for phagophore initiation and cargo engulfment. High‐affinity interactions that “solidify” the receptor on the cargo block phase separation and halt the entire autophagic process [54]. Thus, in many cases, autophagy is likely not a simple recruitment of machinery to a static cargo‐receptor complex. Instead, it is a dynamic self‐organization process, where regulated phase separation(s) on the cargo surface create a physical platform to nucleate the degrading membrane. Intriguingly, this model seems to apply universally to selective autophagy, as these initiation hubs have been observed on a diverse array of cargoes—both native (condensates and organelles) and engineered—in two evolutionarily distant organisms.

### Interplay between UPS and autophagy at the endoplasmic reticulum

The spatial coordination of UPS and autophagy is particularly evident at the ER, where folding demand is exceptionally high and proteostasis failure has immediate pathological consequences. Because the ER lacks intrinsic proteolytic machinery, aberrant proteins must be individually extracted for proteasomal degradation or rerouted to the lysosome when they form larger aggregates. The ER therefore represents a paradigmatic site where proteasomal degradation and selective autophagy intersect (Fig. 2B).

#### ER‐associated degradation (ERAD): First‐line proteasomal disposal

Misfolded proteins within the ER lumen or membrane are initially recognized by ER‐resident chaperones, including BiP, calnexin, and calreticulin, which attempt to refold them or, if folding fails, target them for destruction. As degradation cannot occur within the ER itself, substrates are retro‐translocated into the cytosol. During this process, they are ubiquitinated by ER membrane‐embedded E3 ligases such as HRD1, AMFR/GP78, RNF185, or MARCH6 [55]. The AAA+ ATPase p97/Cdc48 then extracts ubiquitinated clients from the membrane and delivers them to the 26S proteasome [56, 57]. Defects at any step of ERAD—from substrate recognition, retro‐translocation, ubiquitination, or proteasomal turnover—are associated with diverse human pathologies, highlighting ERAD's role as a central proteostasis safeguard.

#### ER‐phagy: Selective autophagy in ER remodeling and clearance

When proteasomal capacity is exceeded, ERAD‐resistant proteins accumulate, or aberrant ER subdomains build up, ER‐phagy provides an adaptive lysosomal route for ER remodeling and clearance. ER‐phagy is mediated by ER‐resident autophagy receptors that directly couple ER membranes to LC3/GABARAP proteins on nascent phagophores through LIRs. Key ER‐phagy receptors exhibit specialized functions: FAM134B regulates ER sheet turnover [23, 58, 59]; RTN3 mediates tubular ER clearance [24]; CCPG1 functions during ER stress recovery [60]; and TEX264 acts as a dominant receptor for nutrient stress‐induced ER‐phagy [61, 62]. Additional receptors such as ATL3, SEC62, and CALCOCO1 further diversify ER‐phagy outputs depending on ER architecture and stress context [63, 64, 65].

Importantly, ER‐phagy is not merely a bulk degradation pathway but serves as a selective quality control mechanism for ER proteostasis, tightly linked to the UPS. As such, ER‐phagy specifically targets ERAD‐resistant aggregates or remodels stressed ER regions when retro‐translocation and proteasomal threading fail. Emerging physiological evidence highlights the importance of this process in secretory tissues, such as protecting chondrocytes from toxic procollagen overload [66, 67, 68]. Thus, ER‐phagy represents a spatially restricted extension of proteostasis control complementing the UPS when proteasomal disposal is saturated or structurally constrained.

#### ER‐to‐lysosome‐associated degradation (ERLAD): Compensatory failsafe pathways

In addition to canonical ER‐phagy, recent work has defined ER‐to‐lysosome‐associated degradation (ERLAD) as a family of context‐dependent compensatory mechanisms that reroute aberrant ER luminal or membrane clients directly to lysosomes when ERAD fails [69, 70, 71]. Mechanistically, ERLAD is highly diverse and can involve micro‐ER‐phagy‐like engulfment, LC3‐dependent vesicular transport, or direct ER–endolysosome communication via membrane contact sites. Unlike classical ERAD, which requires cytosolic extraction, ERLAD enables disposal of clients that are aggregation‐prone, polymeric, or retro‐translocation resistant. Emerging evidence suggests that ERLAD pathways often employ ER chaperones to relay misfolded clients onto ER‐phagy receptors, thereby coupling luminal recognition to export toward the lysosome. Remarkably, ERLAD exit routes frequently repurpose specialized ER subdomains, including ER exit sites [72], ER–lysosome membrane contact sites [71], or three‐way tubular junction hubs to function as ER proteostasis centers [73]. Disease‐relevant substrates cleared through ERLAD include procollagen aggregates [70], α1‐antitrypsin Z polymers [69, 71], mutant proinsulin Akita, and misfolded neuroendocrine prohormones trafficked through specialized ERLAD sites [73, 74, 75].

Notably, some substrates are partitioned between ERAD and ERLAD in parallel rather than sequentially. For example, the Niemann–Pick mutant NPC1 I1061T undergoes both MARCH6‐dependent ERAD and FAM134B‐mediated lysosomal delivery [76], illustrating that proteasomal and lysosomal routes cooperate as integrated safeguards rather than simple backup systems.

#### How do cells sense that proteasomal capacity is exceeded and switch to ERLAD/ER‐phagy?

This remains a central question in ER proteostasis research and has yet to be definitively resolved. However, emerging evidence suggests several interconnected mechanisms. An important pathway involves the unfolded protein response (UPR) activated by sustained ER stress due to the accumulation of misfolded proteins in the ER lumen. The UPR transcriptionally upregulates ER‐phagy receptors such as CCPG1 and FAM134B [60]. At the same time, ER stress causes mTOR inhibition, leading to autophagy induction and activation of kinases, such as CK2 [77]. CK2 is known to phosphorylate the ER‐phagy receptor FAM134B [77, 78]. Phosphorylation is a prerequisite for ubiquitination of FAM134B, receptor clustering and ER‐phagy flux [77, 79]. Thus, a combination of stress kinase signaling, transcriptional reprogramming, and ubiquitination events ensures that ER‐phagy is engaged when proteasomal clearance becomes limiting.

### Proteotoxic stress in the cytosol

While ERAD, ER‐phagy, and ERLAD exemplify compartment‐specific proteostasis control, similar principles govern cytosolic protein quality control, where spatial organization, condensate formation, and degradation pathway switching dictate the cellular response to misfolded proteins.

Aggregation‐prone or intrinsically disordered proteins such as α‐synuclein, Tau, huntingtin, and TDP‐43 can undergo aberrant phase transitions and form cytosolic inclusions that perturb proteostasis, trigger oxidative stress, activate inflammatory signaling, and broadly impair cellular function [80, 81, 82]. In response, cells engage coordinated stress programs that transcriptionally induce chaperones, proteasome components, and autophagy regulators, thereby expanding overall degradative capacity [2, 83]. At the protein level, molecular chaperones and the UPS constitute the first line of defense, attempting substrate refolding or proteasomal elimination of soluble misfolded species. However, once aggregates exceed proteasomal capacity or become structurally resistant to unfolding and threading, selective autophagy pathways—particularly aggrephagy—become essential for clearance of larger assemblies [13, 84, 85].

Aggrephagy relies on ubiquitin‐dependent cargo recognition and autophagy receptors such as p62/SQSTM1, NBR1, OPTN, and TAX1BP1, which couple ubiquitinated aggregates to LC3‐positive membranes [86, 87]. This pathway is strongly upregulated transcriptionally by stress‐responsive regulators including TFEB/TFE3, NRF1, ATF4, and HSF1, linking aggregate burden to lysosomal biogenesis, proteasome recovery, and adaptive proteostasis remodeling [88, 89, 90].

Several comprehensive reviews have covered cytosolic aggregate clearance and its relevance for neurodegeneration in detail [91, 92, 93], highlighting that the UPS–autophagy network must continuously balance rapid proteasomal disposal of soluble clients with autophagic sequestration of phase‐separated and aggregation‐prone assemblies.

## Temporal regulation by stress signaling and network reprogramming

The balance between the UPS and autophagy is not static but is continuously rewired by stress signaling pathways and metabolic state. Coordination occurs at multiple regulatory layers, including transcriptional programs driven by stress‐responsive factors such as ATF4, NRF1/2, TFEB, and HSF1 [2, 88, 89], translational control through the integrated stress response (ISR) [94], and post‐translational mechanisms that directly modulate ubiquitin ligases, autophagy receptors, and proteasome function.

Importantly, when the capacity of one degradative arm becomes limiting, the other can compensate, ensuring proteostasis resilience under fluctuating demands. Two emerging stress paradigms—metabolic limitation and spliceosome dysfunction—illustrate how temporal signaling inputs can reshape degradation network output across subcellular compartments.

### Metabolic control of proteasome localization and proteolytic flux

Metabolic stress provides one of the most direct physiological contexts in which proteasome and autophagy activities must be rapidly coordinated. Proteasomes are dynamically distributed between the nucleus and cytoplasm, and their localization is increasingly recognized as an adaptive mechanism to balance regulatory degradation with nutrient recycling [95,96].

During amino acid scarcity, proteasomal degradation becomes a critical source of intracllular amino acid replenishment, sustaining translation and cellular viability. Indeed, proteasome inhibition under nutrient deprivation markedly impairs protein synthesis, underscoring its role as a metabolic recycling system [97,98]. Recent work by Livneh and colleagues demonstrated that aromatic amino acids (tyrosine, tryptophan, phenylalanine) can induce nuclear sequestration of proteasomes, prevent their cytosolic recycling function, and thereby block amino acid recovery [95]. Exploiting this metabolic vulnerability, forced nuclear confinement of proteasomes selectively impaired highly nutrient‐dependent cancer cells, triggering apoptotic programs and suppressing tumor growth in xenograft models. These findings highlight proteasome spatial redistribution as a metabolic control node and suggest that therapeutic strategies may emerge not only from inhibiting proteasome catalysis but from manipulating its subcellular compartmentalization.

Proteasome relocalization is also observed in other metabolic stress states, including hypoxia [12] a defining feature of solid tumors. Together, these studies reinforce the concept that proteasomal function is deeply integrated with metabolic signaling, spatial proteostasis organization, and cellular fitness. Notably, these metabolic effects intersect with the newly described “degradation hot spots” in both the nucleus and cytosol [51] suggesting that proteasome localization, condensate biology, and metabolic stress responses converge to determine proteolytic output in cancer‐relevant contexts.

### Cryptic splicing‐induced proteotoxicity: Coordinated clearance across cytosol and ER

A second emerging paradigm of temporal network rewiring arises from defects in RNA splicing, which can generate aberrant polypeptides and proteotoxic stress. Impaired spliceosome assembly promotes cryptic splicing and accumulation of misfolded proteins that overwhelm both cytosolic and ER proteostasis systems, thereby linking nuclear gene expression defects directly to protein degradation network activation [99].

Mechanistically, spliceosome dysfunction leads to the production of unstable, aggregation‐prone proteins which become heavily ubiquitinated and form cytosolic inclusions. Simultaneously, misfolded secretory and membrane proteins accumulate within the ER lumen, triggering ER stress and activation of UPR pathways. If unresolved, this dual‐compartment proteotoxicity can drive apoptosis and contribute to the pathogenesis of spliceosome‐associated diseases (spliceosomopathies) [99].

Remarkably, cells respond by engaging both major degradation arms in parallel: the UPS is upregulated to eliminate ubiquitinated cytosolic clients, while FAM134B‐dependent ER‐phagy is activated to selectively clear aberrant ER domains and luminal aggregates. Thus, cryptic splicing stress provides a compelling example of coordinated proteostasis enforcement across cytosol and ER through simultaneous engagement of proteasomal degradation and selective autophagy.

## Future perspectives and therapeutic implications

The UPS and autophagy are increasingly recognized not as parallel degradation routes but as an integrated and adaptive proteostasis network, continuously shaped by ubiquitin signaling, chaperone triage, spatial compartmentalization, and stress‐dependent rewiring.

A major challenge for the field will be to decipher how complex ubiquitin architectures—including mixed or branched chains—and their dynamic editing are interpreted within distinct proteolytic microenvironments to determine cargo fate. Equally important is understanding how shuttle factors and autophagy receptors act as molecular decision nodes whose post‐translational modifications, oligomerization, and condensate partitioning can redirect substrates between proteasomal degradation and selective autophagy. Emerging work on phase‐separated degradation hubs, proteasome storage granules, and autophagy initiation platforms suggests that proteolysis is increasingly organized into localized reaction centers rather than diffusely distributed throughout the cell. At the ER, layered pathways such as ERAD, ER‐phagy, and ERLAD form a continuum of safeguards; yet how substrates are partitioned across these routes and how ER subdomains coordinate lysosomal export remain key open questions. Importantly, metabolic limitation, hypoxia, and cryptic splicing‐induced proteotoxicity highlight that proteostasis control is temporally reprogrammed across compartments rather than globally activated.

Therapeutically, these insights suggest that restoring balanced network function may require targeting specific control nodes—such as proteasome localization, receptor phase behavior, or organelle‐specific autophagy pathways—rather than broadly inhibiting or stimulating degradation. Future therapeutic strategies might therefore focus, for example, on “spatial steering” of the proteasome rather than inhibiting its “engine”. “Traffic control” drugs could exploit metabolic vulnerabilities to sequester the entire degradative machinery into compartments where they are either lethal (in cancer) or protective (in neurodegeneration). Deciphering the organizing principles of UPS–autophagy integration will ultimately enable more selective strategies to counteract proteostasis collapse in aging, neurodegeneration, cancer, and metabolic disease.
